# Safety of repeated neuromodulation by transcranial direct current stimulation (tDCS) in dementia: a narrative review

**DOI:** 10.1192/j.eurpsy.2023.569

**Published:** 2023-07-19

**Authors:** A. A. Daniel, S. De Souza

**Affiliations:** ^1^Medicine, University Of Bristol, Bristol; ^2^Somerset NHS Foundation Trust, Taunton, United Kingdom

## Abstract

**Introduction:**

Transcranial direct current stimulation (tDCS) is a form of neuromodulation most commonly used in depression. tDCS aims to modulate cortical activity by the application of a weak electrical current to the brain via electrodes placed on the scalp. Several studies have identified the potential of tDCS for managing behavioural and psychological symptoms in a range of dementias, including Alzheimer’s disease, vascular dementia, dementia with Lewy bodies and frontotemporal dementia. Although the preliminary data on efficacy is promising, the safety of repeatedly neuromodulating the brain of a person with dementia, by tDCS, has not been extensively reported.

**Objectives:**

Our aim was to review the current literature on how safe it is to repeatedly neuromodulate a brain with dementia.

**Methods:**

Advanced literature searches of PubMed and the Web of Science Core Collection were conducted to identify relevant publications. The search terms deployed were: “tDCS” or “transcranial direct current stimulation” and “frontotemporal dementia” or “vascular dementia” or “Lewy body” or “Alzheimer’s disease”. The following inclusion criteria were applied to the search: (1) publications which focused on the use of tDCS in patients with either frontotemporal dementia, vascular dementia, dementia with Lewy bodies or Alzheimer’s disease, (2) studies involving human participants and, (3) publications written in, or readily translated to English.

**Results:**

216 articles were returned in the initial search. Following the removal of 54 duplicate articles, the remaining 162 underwent eligibility screening using the titles and abstracts. 31 articles were then selected for a full text reading and following this, 12 studies were selected to be included in the review. Across all 12 studies, 3590 sessions of active tDCS were performed with no severe adverse effects being reported. The most commonly occurring adverse effect was a tingling/burning sensation underneath the electrodes, followed by headache and skin changes. These reported effects tended to be mild and short lived.

**Conclusions:**

Overall, the results of the reviewed papers suggest that repeated neuromodulation by tDCS can be safely performed in dementia patients. More and larger studies should aim to perform a greater number of sessions of tDCS, across a longer time period. Few studies assessed for potential brain damage as a result of tDCS and future studies should consider using MRI or monitoring biomarkers to further investigate this.

**Disclosure of Interest:**

None Declared

